# Takayasu arteritis: Diagnosis of a rare clinical entity: A case report

**DOI:** 10.1097/MD.0000000000042096

**Published:** 2025-04-04

**Authors:** Selemon Hileeyesus, Abilo Tadesse, Addissie Fekrie, Berhanu Shetie, Nebiyu Bekele, Guadie Beyazn, Temesgen Tadesse, Weynishet Kebede

**Affiliations:** a Department of Neurology, School of Medicine, College of Medicine and Health Sciences, University of Gondar, Gondar, Ethiopia; b Department of Internal Medicine, School of Medicine, College of Medicine and Health Sciences, University of Gondar, Gondar, Ethiopia; c Department of Radiology, School of Medicine, College of Medicine and Health Sciences, University of Gondar, Gondar, Ethiopia.

**Keywords:** ACR classification criteria, Ishikawa diagnostic criteria, rare disease, Takayasu arteritis

## Abstract

**Rationale:**

Takayasu arteritis is a rare, autoimmune, granulomatous inflammatory vascular disease of the aorta and its main branches. Delayed diagnosis was attributed to vague early clinical features, lack of specific diagnostic marker, rarity of disease, and lack of awareness by physicians of the disease condition.

**Patient concerns:**

A 28-year-old woman presented with sudden onset right sided body weakness associated with transient loss of consciousness, left-sided facial deviation, and slurring of speech. She had history of limb claudication. She was told to have rheumatic regurgitant aortic valve disease 5 years back. Physical examination revealed elevated blood pressure, reduced left radial artery pulse, faintly palpable right dorsalis pedis and posterior tibial arteries pulse, blood pressure asymmetry in arms, early diastolic murmur at erb’s area, and right-sided hemiparesis with ipsilateral supra-nuclear facial palsy. Laboratory markers revealed high erythrocyte sedimentation rate. Two dimensional-transthoracic echocardiography and neck Doppler ultrasound revealed aortic regurgitation, and stenosed and thrombosed left common carotid artery, respectively. Post-contrast computed tomography showed thickened, occluded and thrombosed left common carotid artery, stenosed right common carotid artery, and thickened, stenosed and calcified descending thoracic aorta and abdominal aorta. Brain magnetic resonance imaging showed left basal ganglia infarction. Diagnosis of thromboembolic stroke secondary to Takayasu arteritis was made.

**Diagnoses:**

Diagnosis of Takayasu arteritis was settled using modified Ishikawa Diagnostic Criteria and American College of Rheumatology Classification Criteria.

**Interventions:**

She was started on glucocorticoids 50 mg po daily for 3 months and tapered by 5 mg po weekly over 3 months, azathioprine 100 mg po daily, aspirin 81 mg po daily, atorvastatin 40 mg po daily, amlodipine 10 mg po daily, and cotrimoxazole 960 mg po trice weekly. Physical therapy was continued.

**Outcomes:**

The patient showed marked symptomatic improvement after 3 months of treatment. She was referred to higher health institution for evaluation of vascular intervention.

**Lessons:**

High index of clinical suspicion is required for early diagnosis of rare diseases to prevent adverse outcomes.

## 1. Introduction

Takayasu arteritis (TAK) is a rare, autoimmune vascular disease characterized by granulomatous inflammation affecting the aorta and its primary branches.^[1]^ The disease is named after Dr Mikito Takayasu, Japanese ophthalmologist, after describing absent radial pulse and fundal arterio-venous anomalies of the disease in a 21-year-old woman in 1905.^[2]^ Although TAK occurs in all age ranges, both sexes, and widespread geographic areas, it is frequently observed in young, Asian women.^[3]^ Diagnosis is challenging and often delayed due to early nonspecific clinical presentation. TAK can manifest incidentally (vascular imaging for other indications) or as complications of affected vessels such as hypertension, transient ischemic attack or ischemic stroke, angina or myocardial infarction, and mesenteric ischemia.^[4,5]^ We used modified Ishikawa Diagnostic Criteria and American College of Rheumatology (ACR) Classification Criteria to reach at a diagnosis.^[6–8]^ Here, we presented a case of TAK in a 28-year-old woman, who developed thromboembolic stroke. The case was discussed with reviewed literatures.

## 2. Case presentation

**Figure 1.**
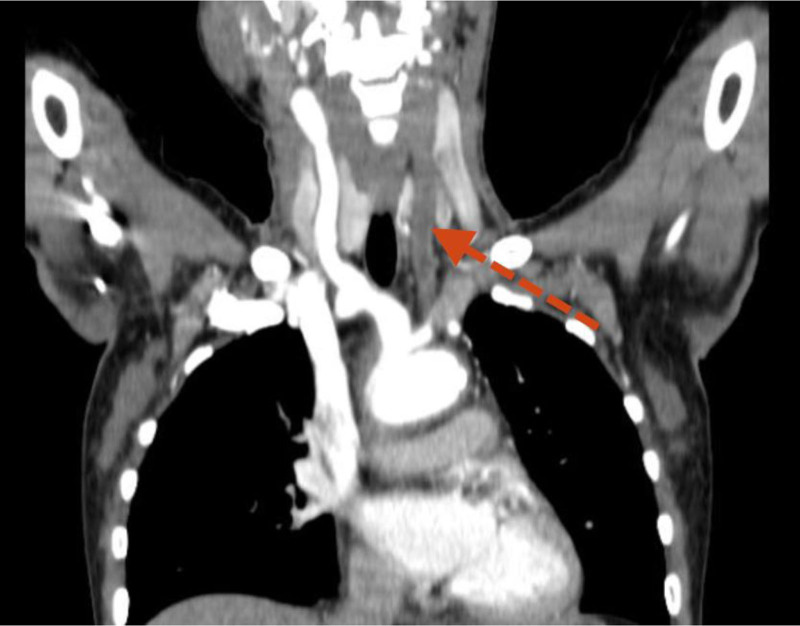
Post-contrast CT scan: thickened, occluded, and thrombosed left common carotid artery (red arrow). Right common carotid artery luminal narrowing and dilatation was visualized. CT scan = computed tomography scan.

**Figure 2.**
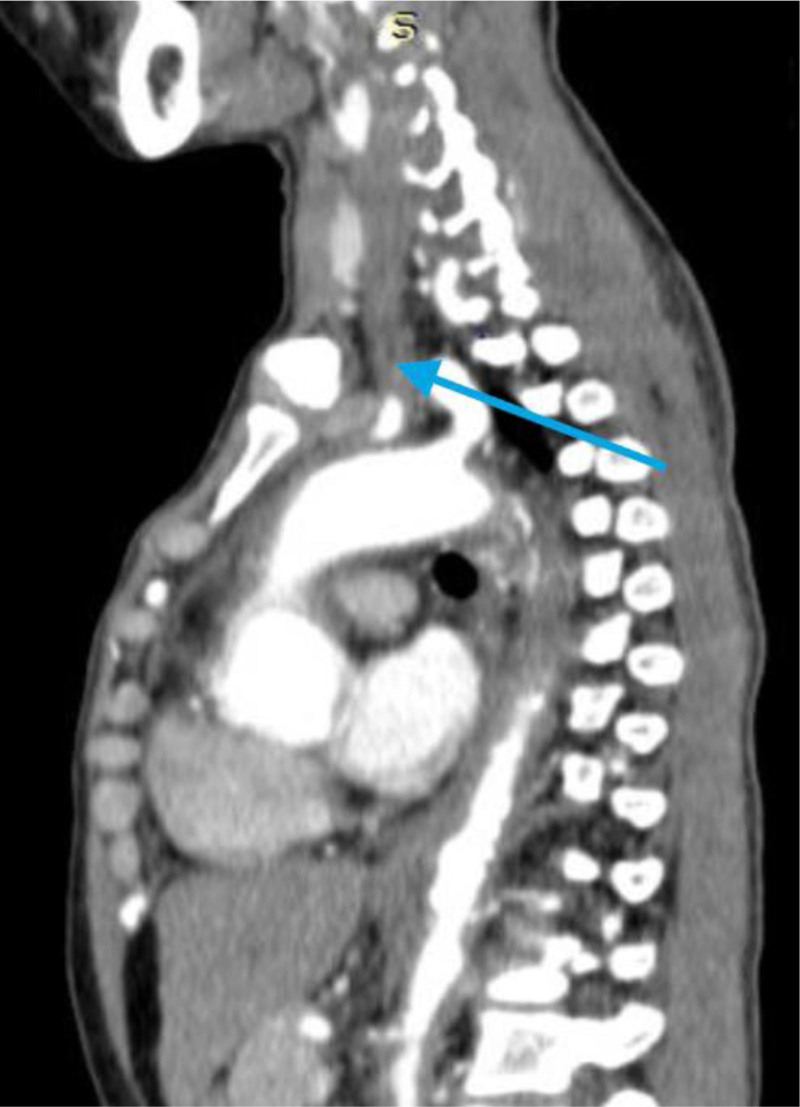
Post-contrast CT scan: thickened, stenosed, occluded, and thrombosed left common carotid artery (blue arrow). Descending thoracic aorta and abdominal aorta wall thickening and luminal narrowing were visualized. CT scan = computed tomography scan.

**Figure 3.**
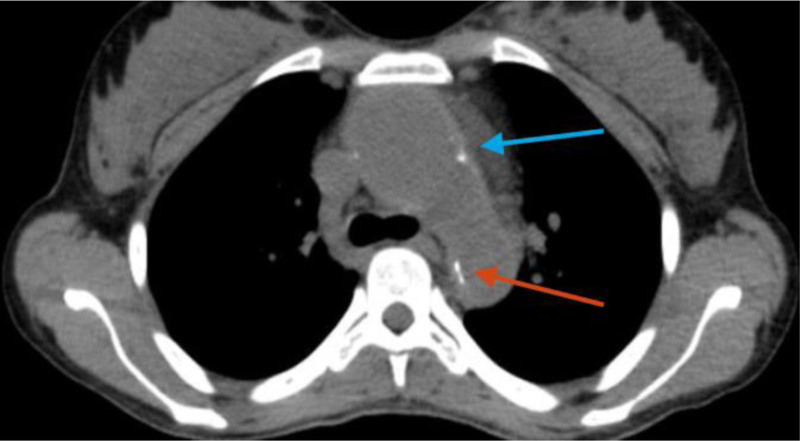
Pre-contrast CT scan: thickened aortic arch wall with luminal dilation, and calcification (blue arrow). Narrowed descending thoracic aorta with calcification (red arrow) was visualized. CT scan = computed tomography scan.

**Figure 4.**
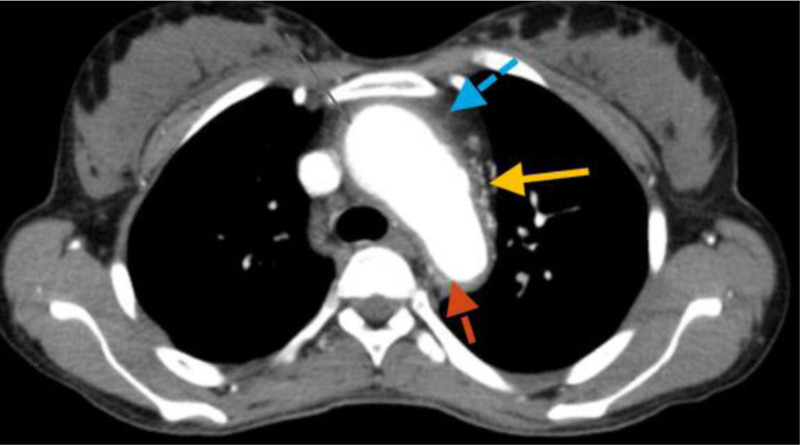
Post-contrast CT scan: Thickened ascending aorta (blue arrow), aortic arch (yellow arrow), and narrowed lumen of descending aorta (red arrow) with calcification. The ascending aorta and aortic arch were dilated. CT scan = computed tomography scan.

A 28-year-old woman presented to University of Gondar hospital with sudden onset right-sided body weakness, and left-sided facial deviation with slurring of speech. She had transient loss of consciousness persisting for 30 minutes, but no headache, vomiting, or episodic abnormal body movements. She was told to have rheumatic regurgitant aortic valve disease 5 years back, and had been on lasix, 20 mg po twice daily, spironolactone 25 mg po daily, and monthly benzathine penicillin. She occasionally felt pain on her left arm while combing her hair. She had history of prolonged low grade fever, unquantified weight loss, and easy fatigability. No history of dyspnea on exertion, angina or palpitation. She had no diplopia, dizziness, tinnitus or syncope. No history of headache, blurring of vision, neck pain or jaw claudication. No history of joint pain, skin rash or oral ulcers. She had no history of hypertension, diabetes, or chronic kidney disease. On physical examination, she was acutely sick looking. Vital signs, blood pressure = 155/96 mm Hg (right arm), 140/87 mmg (left arm); pulse rate = 80 beats per minute; respiratory rate = 24 breaths per minute; Temperature^0^ = 36 °C, pulse oxymeter saturation = 93% at ambient air. She had moderate pallor of conjunctivae and non-icteric sclerae. Chest was resonant to percussion and clear to auscultation. On cardiovascular examination, pulse volume was weaker on the left radial artery. The right dorsalis pedis and posterior tibial arteries were faintly palpable. There was early diastolic murmur at erb’s point. Audible bruits were appreciated over both common carotid arteries. There were no organomegaly or signs of ascites, and aortic or renal bruits were not appreciated on abdominal examination. On neurological examination, she was oriented in time, place and person. NIHSS score was 6 to 7. She had left-sided facial deviation, while other cranial nerves were intact. She had right-sided hemiparesis, but had no sensory loss. Meningeal irritation signs were negative. On laboratory investigation, white blood cells = 6500/µL (neutrophils 61%, lymphocytes = 31%) (normal range 4.5–11 × 10^3^/µL), hemoglobin = 9.2 g/dL (normal range = 12–16 g/dL), platelets = 252,000/µL (normal range = 150–450 × 10^3^/µL) erythrocyte sedimentation rate (ESR) = 94 mm in first hour (normal range ≤ 20 mm/h). Serum liver biochemical tests, serum renal function tests, and serum electrolytes were within normal limits. Serological tests for human immunodeficiency virus, hepatitis B surface antigen, and anti-hepatitis C virus antibody were negative. Serum anti-nuclear antibody, rheumatoid factor, and venereal disease research laboratory tests were negative. Trans-thoracic echocardiography showed mild aortic regurgitation and mild aortic root dilation. No mitral valve morphologic abnormalities. No abnormalities on left ventricular size and contractility. Ejection fraction was 60%. Carotid Doppler study revealed diffuse thickening of both common carotid arteries, and hypo-echoic filling defect of the left common carotid artery along its length with caliber stenosis. Abdominal ultrasound showed normal sized kidneys and echo texture. No abnormalities were detected on abdominal aorta and renal arteries. Post-contrast chest and abdominal computed tomography scan revealed left common carotid artery occlusion throughout its length and thrombus formation (Figs. 1 and 2). Ascending aorta and aortic arch were dilated. Descending thoracic aorta and abdominal aorta had wall thickening and luminal narrowing with calcification (Figs. 3 and 4). Brain magnetic resonance imaging detected T1 hypointense, and T2 and fluid attenuated inversion recovery hyperintense lesion in the left basal ganglia, which suggested left basal ganglia infarction. Diagnosis of thromboembolic stroke secondary to Takayasu arteritis was made. Diagnosis of Takayasu arteritis was made based on modified Ishikawa Diagnostic Criteria, which fulfilled characteristic signs and symptoms (limb claudication, pulse differences in limbs, systolic blood pressure difference > 10 mm hg in limbs, and fever) as major criteria; and high ESR, hypertension, aortic regurgitation (using clinical and echocardiography), left common carotid artery lesion, descending thoracic aorta lesion, and abdominal aorta lesion as minor criteria (required for diagnosis: 2 major, 1 major and 2 minor, or 4 minor). In addition, the patient was classified as having Takayasu arteritis based on The American College of Rheumatology Classification Criteria, which fulfilled age at diagnosis < 40 years, limb claudication, blood pressure difference > 10 mm Hg between arms, reduced left radial pulse, and vascular abnormalities on imaging (required for ACR classification: at least 3 of the 6 criteria). She was initiated on prednisolone 50 mg po daily for 3 months and tapered by 5 mg per week over 3 months, azathioprine 100 mg po daily, aspirin, 81 mg po daily, atorvastatin 40 mg po daily, amlodipine 10 mg po daily, and cotrimoxazole 960 mg po trice weekly. Physical therapy was continued. She had marked symptomatic improvement after 3 months of treatment. She was referred to higher health institution for evaluation of vascular intervention.

## 3. Discussion

TAK is a chronic, autoimmune, granulomatous large vessel vasculitis, which primarily affects the aorta and its main branches.^[1]^ Global annual incidence of TAK is ~0.4 to 2.6 per million people. It is present worldwide, but commonly noticed in Asian population.^[3]^ Etiology of TAK remains unknown, but infection, autoimmunity and genetic factors play a role in its pathogenesis. Immune intolerance to pathogens or self-antigens residing in immune-privileged site (elastic media) of large vessels might trigger chronic vascular inflammation. The arterial wall inflammation and endothelial damage result in various vascular complication, including stenosis, occlusion, thrombus formation, and aneurysmal dilatation. The clinical presentation of TAK progresses in 3 phases. In early systemic inflammatory phase (pre-pulseless phase), it is predominated by nonspecific constitutional symptoms including fever, night sweats, weight loss, arthralgia, and fatigability for weeks to months. If left uncontrolled, the vascular inflammatory phase (pulseless phase) supervenes, and characterized by limb claudication, carotidynia, reduced arterial pulse, and asymmetric blood pressure. The last “fibrotic” or “burnt-out” phase is defined by significant vascular occlusion and manifests with ischemic stroke, myocardial infarction, limb ischemia or gangrene, amaurosis (vision loss), hypertension, or mesenteric ischemia.^[4,5]^ Diagnosis of TAK is delayed in years due to nonspecific early clinical symptoms, lack of diagnostic marker, rarity of disease, and lack of awareness by physicians of the disease condition. Diagnostic criteria comprising clinical, laboratory tests, and vascular imaging modalities were developed by Ishikawa in 1988, and later revised by Sharma et al in 1995.^[6,7]^ It comprised 3 major criteria (left subclavian artery lesion, right subclavian artery lesion, and characteristic symptoms and signs) and 10 minor criteria (high ESR, carotidynia, hypertension, aortic regurgitation, pulmonary artery lesion, left common carotid artery lesion, brachiocephalic trunk lesion, descending thoracic aorta lesion, abdominal aorta lesion, and coronary artery lesion). Presence of 2 major, 1 major and 2 minor, or 4 minor criteria suggests high probability of TAK. The patient was diagnosed to have Takayasu arteritis, since she had 1 major (characteristic symptoms and signs: limb claudication, pulse difference in limbs, blood pressure asymmetry) and 6 minor criteria (high ESR, aortic regurgitation, hypertension, left carotid artery lesion, descending thoracic aorta lesion, and abdominal aorta lesion). Classification criteria were developed in 1990 by ACR, which comprised age of disease onset < 40 years, claudication of extremities, decreased brachial artery pulse, blood pressure difference > 10mmHg, bruit over the subclavian or aorta, and angiogram abnormalities. The presence of 3 or more criteria classifies as TAK.^[8]^ The patient contented the 4 criteria including age at onset < 40 years, limb claudication, blood pressure asymmetry, and angiogram abnormalities. Decreased left radial artery pulse, faintly palpable right dorsalis pedis and posterior tibial arteries pulse, and bruits over both common carotid arteries could be attributed to TAK. Vascular imaging modalities of choice include computed tomography angiography, magnetic resonance angiography, fluoro-deoxy-glucose-positron emission tomography, and Doppler ultrasound. These imaging modalities reveal wall thickening, luminal stenosis and occlusion, thrombus formation, and aneurysmal dilatation of the affected large vessels and its primary branches. Numano system was developed in 1996 to depict topography of arterial lesions.^[9,10]^ Our patient was classified as type V (type IIb + type IV) based on angiographic classification. Inflammatory markers including ESR and C-reactive protein are used to monitor disease activity, even though both markers are poor indicators of disease activity. They are elevated in half of inactive vascular lesion, and are at normal level in one-third of active vascular disease.^[4,5]^ Possible differential diagnoses for TAK include Giant cell arteritis, fibromuscular dysplasia, and atherosclerotic vascular disease. Giant cell arteritis preferably involves external carotid artery. It usually occurs in elderly women, and often presents with headache, pain over the temples, scalp tenderness, visual disturbance, and jaw claudication. Diagnosis is confirmed by biopsy of the affected temporal artery. Fibromuscular dysplasia is congenital blood vessel wall abnormalities. It frequently affects middle-aged women. It involves any artery of the body, but frequently affects renal and carotid-vertebral arteries. It clinically presents as asymptomatic (incidental finding on vascular imaging), hypertension in renal arterial disease, or stroke in carotid-vertebral arterial disease. The diseased arterial territories could be complicated by aneurysms, dissections and occlusions. Diagnosis is made by angiographic imaging, which reveals “string of beads” appearance of the involved arterial bed. Lastly, atherosclerotic vascular disease is other clinical mimicker of TAK. It is focal and segmental stenotic lesions of arteries caused by fibrous plaque (atheroma). In this case, younger age at diagnosis and absence of predisposing conditions makes atherosclerosis as unlikely diagnosis.^[4,5]^ Medications for TAK include glucocorticoids, synthetic disease modifying anti-rheumatic diseases (DMARDs) (azathioprine, methotrexate, leflunomide, mycophenolate mofetil, cyclophosphamide), and biologic DMARDS (TNF inhibitors, tocilizumab). Combinations of glucocorticoids and synthetic DMARDS are initiated as initial therapy of choice to address steroid-sparing regimen and persistent vascular inflammation. Biologic DMARDS are preserved for relapsing or refractory cases, and in aggressive clinical course.^[11–13]^ Invasive interventions including transluminal angioplasty, stent placement and bypass grafting are reserved for severe symptomatic coronary artery or cerebrovascular disease, uncontrolled hypertension from renal artery stenosis, severe aortic regurgitation or coarctation, critical limb ischemia from stenotic or occlusive lesions, and aneurysm at risk of rupture.^[14]^ The patient was started on medications (high-dose prednisolone, azathioprine, aspirin, atorvastatin, amlodipine, and cotrimoxazole) and physical therapy, and showed marked symptomatic improvement after 3 months of treatment. She was referred to higher health institution for evaluation of vascular intervention.

## 4. Conclusion

Diagnosis of Takayasu arteritis was made using modified Ishikawa Diagnostic Criteria and American College of Rheumatology Classification Criteria. Delayed diagnosis was attributed to early vague clinical features, lack of specific diagnostic marker, rarity of disease, and lack of awareness by physicians of the disease condition.

## Acknowledgments

We are grateful to the medical personnel who were caring for the patient.

## Author contributions

**Conceptualization:** Selemon Hileeyesus, Abilo Tadesse.

**Data curation:** Selemon Hileeyesus, Abilo Tadesse.

**Investigation:** Selemon Hileeyesus.

**Supervision:** Abilo Tadesse, Nebiyu Bekele, Guadie Beyazn, Weynishet Kebede.

**Validation:** Selemon Hileeyesus, Abilo Tadesse, Addissie Fekrie, Berhanu Shetie, Nebiyu Bekele, Guadie Beyazn, Temesgen Tadesse, Weynishet Kebede.

**Visualization:** Selemon Hileeyesus, Abilo Tadesse, Addissie Fekrie, Berhanu Shetie, Nebiyu Bekele, Guadie Beyazn, Temesgen Tadesse, Weynishet Kebede.

**Writing – original draft:** Selemon Hileeyesus, Abilo Tadesse.

**Writing – review & editing:** Selemon Hileeyesus, Abilo Tadesse, Addissie Fekrie, Berhanu Shetie, Nebiyu Bekele, Guadie Beyazn, Temesgen Tadesse, Weynishet Kebede.
